# Editorial: Perspectives in virus and host: 2025

**DOI:** 10.3389/fcimb.2026.1849483

**Published:** 2026-04-22

**Authors:** Yu Yang, Severino Jefferson Ribeiro da Silva, Dhaneshwar Kumar, Kanchan Bhardwaj, Yong Qi

**Affiliations:** 1 Huadong Research Institute for Medicine and Biotechniques, Nanjing, Jiangsu, China; 2 Department of Pharmaceutical Sciences, Leslie Dan Faculty of Pharmacy, University of Toronto, Toronto, ON, Canada; 3 Immunoregulation Section, Kidney Diseases Branch, National Institute of Diabetes and Digestive and Kidney Diseases, NIH, Bethesda, MD, United States; 4 Department of Biotechnology, Manav Rachna International Institute of Research Studies, Faridabad, Haryana, India; 5 Regional Centre for Biotechnology, NCR Biotech Science Cluster, Faridabad, Haryana, India; 6 School of Engineering, China Pharmaceutical University, Nanjing, China; 7 School of Life Science, Xuzhou Medical University, Xuzhou, China

**Keywords:** emerging viral threats, global public health, host-directed therapy, viral pathogenesis, virus-host interactions

## 1 Introduction

The persistent global burden of viral disease is driven by the rapid evolution of pathogens—including hepatitis B, hepatitis C, SARS-CoV-2, influenza viruses, respiratory syncytial virus (RSV), and emerging zoonotic agents—and their ability to evade or subvert host immunity. As climate change and ecological disruptions expand zoonotic reservoirs, translating fundamental molecular insights into clinical interventions becomes increasingly critical. This Research Topic, *Perspectives in Virus and Host: 2025* compiles 19 studies that interrogate these complex virus-host dynamics across the entire infection lifecycle. Moving beyond descriptive biology, these contributions aim to elucidate mechanisms of virus-induced pathology, develop innovative experimental models, and identify novel therapeutic vulnerabilities. Ultimately, by integrating fundamental virological research with clinical applications, this Research Topic establishes a robust translational framework essential for anticipate, manage, and mitigate future viral outbreaks.

## 2 Mechanistic insights into virus-host interplay and host-directed therapeutic targets

The general concept of entry for hepatitis B, hepatitis C and herpes simplex virus type 1 (HSV-1) is that they enter host cells through distinct receptor-mediated processes. The hepatitis B virus targets hepatocytes by binding to the NTCP receptor, leading to receptor-mediated endocytosis, fusion, and delivery of its DNA to the nucleus. The hepatitis C virus uses a multi-step entry process involving receptors such as CD81, SR-BI, claudin-1, and occludin, followed by clathrin-mediated endocytosis and endosomal fusion to release its RNA into the cytoplasm. In contrast, HSV-1 typically enters via direct membrane fusion after binding to receptors like nectin-1 or HVEM, allowing the capsid to travel to the nucleus, where viral DNA is released.

Our Research Topic highlights a novel mechanistic link between viral entry and downstream pathophysiology. For instance, chronic infection with hepatitis B and C viruses precipitates diabetes-like metabolic dysregulation by orchestrating a *STAT3*-centered “inflammation-oxidative stress-epigenetics” axis. This synergistic network links hepatic pathology to systemic metabolic dysfunction, underscoring the potential of multi-target therapeutic strategies that address both liver disease and diabetic sequelae (Wang et al.). In contrast, HSV-1 infection exploits the aryl hydrocarbon receptor (*AhR*) as a proviral mediator by triggering a kynurenine-dependent activation loop. Upon activation, *AhR* assembles into a transcription complex with RNA polymerase II (RNAPII) to drive the expression of both viral immediate-early genes and entry receptors. This process establishes a pro-transcriptional environment that sustains robust lytic replication and identifies the *AhR* signaling axis as a promising target for host-directed antiviral intervention (Huang et al.).

The transcriptional landscape of SARS-CoV-2 infection is governed by a coordinated network of inflammatory regulators, antiviral mediators, metabolic modulators, and stress-responsive elements. Together these factors regulate the signaling pathways that govern metabolic adaptation, immune regulation, and inflammation in response to pathogen-associated molecular patterns (PAMPs) and damage-associated molecular patterns (DAMPs). The authors highlight how transcriptional networks and post-translational modifications can determine host-virus dynamics affecting immune response and disease progression (Modipane et al.). In the context of RSV infection, host transcriptional responses in HEp-2 cells reveal significant upregulation of genes involved in cell-cycle arrest (*CDKN1A*), cytoskeletal reorganization (*vimentin*), apoptosis modulation (*MCL1*), immune evasion (*CD59*), and inflammation (*IL-6*). These findings delineate the key epithelial response to RSV infection and identify these specific regulatory hubs as prime candidates for early-stage therapeutic intervention aimed at disrupting viral subversion of host pathways (Pastey et al.). Krüppel-associated box-associated protein 1 (*KAP1/TRIM28*) demonstrates dual functionality in antiviral immunity, acting both as a transcriptional repressor that facilitates viral silencing through heterochromatin formation and as a modulator of innate immune signaling pathways via dynamic post-translational modifications. This duality highlights its context-dependent roles across diverse viral families and helps resolve longstanding mechanistic ambiguities. (Xin et al.). Similarly, *SERINC5* functions as a broad-spectrum restriction factor, inhibiting both retroviruses and non-retroviruses by blocking membrane fusion and attenuating viral infectivity, thereby reinforcing host defense at the plasma-membrane level and expanding the toolkit of host-directed antiviral factors with potential for pan-viral applications (Yu et al.). Host-dependent C-to-U RNA editing in SARS-CoV-2 generates novel out-of-frame *AUG* codons that generate putatively functional open reading frames with optimized codon adaptation indices, Kozak scores, and tRNA adaptation indices, conferring selective advantages that are under positive evolutionary pressure in circulating viral populations (Zhang et al.). Finally, myeloid cell leukemia-1 (*Mcl*-1) acts as a host antiviral restriction factor whose knockout or knockdown markedly enhances replication of both RSV and influenza A virus in mouse embryonic fibroblasts and human alveolar epithelial cells, while also increasing syncytia formation and late-stage apoptosis, underscoring its protective role during respiratory viral infection and raising important cautions for the clinical use of *Mcl*-1 inhibitors in oncology where such agents may inadvertently heighten viral susceptibility (Prescott et al.). Collectively, this Research Topic identifies novel host-directed therapeutic targets and advances our understanding of the molecular mechanisms governing infection outcomes.

## 3 Innovating experimental models and technical advances

Bridging the gap between biosafety constraints and physiological fidelity remains a central challenge in virology. Recent advancements in three-dimensional (3D) organ-tissue mimetics, utilizing DNA origami and VLP-based tracking, now enable detailed interrogation of H5N1-glycocalyx interactions in a high-throughput, low-containment setting (Hassan et al.). These approaches provide more accessible yet biologically meaningful platform for studying viral entry and host interactions. Emerging perspectives on alphavirus biology highlight a cooperative model of encapsidation, assembly, and budding, in which capsid proteins and multiple high-affinity genomic RNA-binding sites act in concert to ensure selective packaging. This dual-model framework supports host-cell-dependent switching between nucleocapsid-directed and glycoprotein-directed budding strategies, challenging the classical single-packaging-signal paradigm and offering important insights for the rational design of next-generation virus-like particle vaccines (Bhardwaj et al.). In parallel, metagenomic profiling of bronchoalveolar lavage fluids from patients with lower respiratory tract infections reveals distinct viral taxonomic signatures associated with severe disease, including enrichment of Phixviricota, Malgrandaviricetes, Petitvirales, and Microviridae lineages. Integration of virus–bacteria co-occurrence networks with clinical indicators, such as lymphocyte counts and C-reactive protein levels, further identifies novel microbial biomarkers that move beyond single-pathogen frameworks toward more comprehensive diagnostics and personalized risk assessment (Zhu et al.). Collectively these technological and conceptual advances overcome key experimental limitations and enable deeper exploration of complex infection dynamics across diverse viral systems, paving the way for more predictive and scalable translational research platforms.

## 4 Advancing novel prevention and treatment strategies with translational potential

Mechanistic insights from these studies directly inform the development of next-generation interventions aimed at addressing current therapeutic gaps. A novel lytic phage (vB_SmaS_QH16) isolated from hospital sewage exhibits a broad host range against multidrug-resistant *Stenotrophomonas maltophilia*, demonstrates robust biofilm inhibition and eradication capabilities. Notably, it lacks virulence or resistance genes, and significantly improves survival in both *Galleria mellonella* and murine infection models, positioning it as a promising candidate for phage therapy and a sustainable alternative for managing nosocomial infections in the era of antibiotic resistance (Cheng et al.). In parallel, integrative proteome-wide structural analysis and high-throughput docking across multiple flaviviruses—including Zika virus, yellow fever virus, West Nile virus, Saint Louis encephalitis virus, and Usutu virus infection—and identify conserved binding pockets in NS5 polymerase and E glycoprotein. These sites are targeted by broad-spectrum scaffolds; with lead compounds such as myricetin and temoporfin exhibiting favorable multitarget profiles and strong binding affinities in the low-micromolar to nanomolar range. This expands therapeutic possibilities beyond virus-specific approaches and supports the development of combination strategies against the broader Flaviviridae family (Soares et al.). Furthermore porcine circovirus type 2 (PCV2) exploits Beclin1-mediated autophagy to enhance its replication in PK-15 cells. Silencing Beclin-1 reduces both autophagic flux and viral replication, whereas its overexpression or pharmacological induction with rapamycin amplifies these effects; conversely, inhibition with MHY1485 reverses them. These findings identify autophagy regulation as a potential therapeutic target for controlling this economically significant pathogen and may also inform similar strategies in human viral infections (Li et al.). When considered alongside the antiviral function of *Mcl*-1, these findings highlight the need for cautious application of autophagy- or apoptosis-modulating therapies in oncology, as such interventions may inadvertently increase susceptibility to respiratory viral infections. This highlights the importance of balanced risk-benefit assessment in patients with comorbid conditions and supports the development of safer, more target therapeutic strategies.

## 5 Integrating ecological evolution, genetics, and public health

Effective viral control requires integrating mechanistic laboratory insights with broader ecological and population-level perspectives. In healthy airways, the respiratory virome is dominated by bacteriophages that help maintain microbial balance and prime host immunity. In contrast disease states are characterized by dysbiosis that contributes to the pathogenesis of conditions such as asthma, chronic obstructive pulmonary disease, pulmonary fibrosis, and pneumonia. These observations challenge the traditional view of viruses solely as pathogens and highlights new avenues for virome-based diagnostics and immunomodulatory therapies (Wang et al.). Genomic surveillance of Getah virus infection has reconstructed its transcontinental transmission across Malaysia, Japan, and China, documents an expanded vertebrate host range that now includes pangolins and squirrels and identifies positive selection signatures in the E2 and NSP1 proteins as the molecular basis for cross-species transmission. This detailed mapping of adaptive evolution highlights the indispensable role of long-term genomic monitoring in anticipating future zoonotic risks (Yuan et al.). Genetic association studies further link the rs2070788 polymorphism in *TMPRSS2* (under a recessive model) to “brain fog” in Long COVID, particularly among Brazilian healthcare professionals, with females and young adults (18–29 years) identified as high-risk groups. In contrast the rs12329760 variant shows no significant association, thereby supplying genetic clues for risk stratification and mechanistic investigation into post-acute sequelae of SARS-CoV-2 (Costa Telles et al.). In a complementary clinical study, approximately 5% of patients with moderate COVID-19 exhibited elevated platelet counts above the normal range accompanied by increased T cell levels. A positive correlation between platelet and B cell counts in these patients suggests a potential role for platelets and their mediators in supporting recovery highlighting an underexplored aspect of host immune regulation (An et al.). At the public health level, awareness and attitudes toward human papillomavirus (HPV) infection and vaccination remain alarmingly poor among medical students in Jordan. Male students are less likely to receive or recommend vaccination (odds ratios 0.61 and 0.62, respectively), emphasizing the need for targeted educational initiatives and public health strategies to improve vaccine uptake across the Middle East and similar resource-limited settings (Taha et al.). Collectively, these findings illustrate that sustainable infection control depends on embedding molecular and mechanistic insights within their epidemiological and social contexts.

## 6 Concluding remarks and future perspectives

The 19 studies in this Research Topic collectively deepen our understanding of virus–host interactions, bridging molecular mechanisms with public health frameworks and accelerating the translation of fundamental discoveries into potential therapeutic strategies. Moving forward, research should prioritize three areas: (1) mapping regulatory networks to identify broad-spectrum antiviral targets, (2) optimizing phage and host-directed therapies for clinical implementation, and (3) integrating genomic and epidemiological data to enable predictive risk modeling. In light of the rapid pace of viral evolution, interdisciplinary collaboration remains essential. By offering novel conceptual frameworks and technical approaches, this Research Topic strengthens the foundation needed to anticipate, prevent and mitigate future viral threats.

